# Retraction Note: Mesenchymal stem cells ameliorate renal fibrosis by galectin-3/Akt/GSK3β/Snail signaling pathway in adenine-induced nephropathy rat

**DOI:** 10.1186/s13287-024-03668-6

**Published:** 2024-02-27

**Authors:** Huajun Tang, Peiyue Zhang, Lianlin Zeng, Yu Zhao, Libo Xie, Bo Chen

**Affiliations:** 1 Department of Human Anatomy, School of Basic Medical Sciences, Southwest Medical University, No.1, Section 1, Lingxiang Road, Matan Long District, Luzhou, Libo Xie, 646000 Sichuan People’s Republic of China; 2 Department of Urology, Sichuan Clinical Research Center for Nephropathy, the Affiliated Hospital of Southwest Medical University, Southwest Medical University, Luzhou, 646000 Sichuan People’s Republic of China

**Retraction Note: Stem Cell Research & Therapy (2021) 12:409**

10.1186/s13287-021-02429-z

RETRACTION NOTE.

The Editors in Chief have retracted this article after concerns were raised about the data reported. Specifically:

- In Fig. 7a, there is evidence of a potential splice between lanes 2 and 3 of GAPDH blot.
- The GAPDH blot of Fig. 11a appears to duplicate the GAPDH blot of Fig. 11c. In Fig. 11c, there is also evidence of a potential splice between lanes 1 and 2 of Galectin 3 blot.
- In Fig. 15, several panels appear to partially or wholly duplicate one another: the TGF-β1 + DMEM/F12-treated subgroup of control and the TGF-β1 + MSCs-CM-treated subgroup of Gal-3 OE (both α-SMA); the MSCs-CM-treated subgroup of control (E-Cadherin) and the normal subgroup of Gal-3OE (α-SMA); and the TGF-β1 + DMEM/F12-treated subgroup of control (E-Cadherin) and the normal subgroup of control (α-SMA).

The Editors in Chief therefore no longer have confidence in the reliability of the data presented. Bo Chen has stated on behalf of all authors that they agree with the retraction.

